# Conducting Environmental Health Research in the Arabian Middle East: Lessons Learned and Opportunities

**DOI:** 10.1289/ehp.1104031

**Published:** 2012-02-22

**Authors:** Karin B. Yeatts, Mohamed El-Sadig, Habiba I. Ali, Fatma Al-Maskari, Alan Campbell, Shu Wen Ng, Lisa Reeves, Ronna L. Chan, Christopher A. Davidson, William E. Funk, Maryanne G. Boundy, David Leith, Barry Popkin, Jacqueline MacDonald Gibson, Ivan Rusyn, Andrew F. Olshan

**Affiliations:** 1 Department of Epidemiology, Gillings School of Global Public Health, University of North Carolina, Chapel Hill, North Carolina, USA; 2 Department of Community Medicine, Faculty of Medicine and Health Sciences, United Arab Emirates University, Al Ain, United Arab Emirates; 3 Department of Nutrition and Health, Faculty of Food and Agriculture, United Arab Emirates University, Al Ain, United Arab Emirates; 4 Collaborative Studies Coordinating Center, Department of Biostatistics, University of North Carolina, Chapel Hill, North Carolina, USA; 5 Department of Nutrition, University of North Carolina, Chapel Hill, North Carolina, USA; 6 Department of Environmental Sciences and Engineering, University of North Carolina, Chapel Hill, North Carolina, USA; 7 Laboratory for Human Biology Research, Department of Anthropology, Northwestern University, Evanston, Illinois, USA

**Keywords:** environmental epidemiology, indoor air, international issues, policy, population health

## Abstract

Background: The Arabian Gulf nations are undergoing rapid economic development, leading to major shifts in both the traditional lifestyle and the environment. Although the pace of change is brisk, there is a dearth of environmental health research in this region.

Objective: We describe challenges and successes of conducting an environmental epidemiologic study in the United Arab Emirates (UAE), a Gulf nation in the Middle East, with an inter-disciplinary team that includes in-country academic and government collaborators as well as U.S. academic collaborators.

Discussion: We present several issues, including study and data collection design, exposure assessment, scheduling and time coordination, quality assurance and quality control, and institutional review board protocols. These topics are considered in a cultural context. Benefits of this research included building linkages among multinational, interdisciplinary team members, generating data for local environmental decision making, and developing local epidemiologic research capacity. The Middle Eastern culture of hospitality greatly benefited the project team.

Conclusion: Cultural differences impact multiple aspects of epidemiologic research and should be respectfully addressed. Conducting international population-based environmental research poses many challenges; these challenges can be met successfully with careful planning, cultural knowledge, and flexibility. Lessons learned are applicable to interdisciplinary research all over the world. The research conducted will benefit the environmental and public health agencies of the UAE and provide the nation’s leadership with country-specific environmental health data that can be used to protect the public’s health in a rapidly changing environment.

In the past 50 years, Arabian Gulf nations have experienced a great increase in wealth from oil, accompanied by rapid urbanization. The United Arab Emirates (UAE) is a prime example of this swift modernization. The UAE is of scientific interest to environmental health researchers for many reasons, including the rapid transformation of its physical environment from desert to cities, with subsequent increases in traffic- and industry-related pollutants; the potential for unique pollutant mixtures; naturally occurring, extremely high temperatures; and rapid changes in lifestyle and dietary patterns. However, little environ-mental health-related research has been published on this region despite accelerated environmental changes.

A recent PubMed search (conducted in December 2010) for “environmental health” and “United Arab Emirates,” “Kuwait,” “Oman,” “Bahrain,” “Saudi Arabia” or “Qatar” (combined population 38 million) yielded < 300 articles, whereas the same search with “United States” (population 307 million) identified 30,000 articles. Thus, on a per capita basis, approximately 12 times as many published articles pertain to environmental health in the United States compared with the Arabian Gulf nations. Similarly, a recent Thomson Reuters global research report (Adams et al. 2011) indicated that for 2005–2009, the Middle East region (Arabian, Persian, and Turkish Middle East) published 47,201 publications in clinical medicine and 4,676 publications in environment/ecology, with 4.37% and 3.24% of the world’s output, respectively, on Thomson Reuters Web of Knowledge^SM^ in these areas (Adams et al. 2011). Annual research publication output for Bahrain, Kuwait, Qatar, Oman, and the UAE was < 1,000 papers per year for these countries in 2009, although growth rates are rising steeply and show a large potential for enhanced scientific research in the region (Adams et al. 2011).

In 2008, the Environment Agency–Abu Dhabi (EAD) undertook an ambitious project to develop an environmental health research and policy agenda to guide the newly forming regulatory and scientific institutions of the UAE. Because it is a young nation (founded in 1971) that had a small population until recently, the UAE had not established a comprehensive environmental health research agenda. This project was intended to provide information toward the development of such agendas for multiple agencies. Collaborating with UAE University, the Gillings School of Global Public Health at the University of North Carolina at Chapel Hill (UNC) was awarded a competitive contract to provide scientific research support for this project. Tasks of the team included *a*) quantifying the environmental burden of disease in the UAE (based on existing data); *b*) reviewing environmental policies and research programs to identify clear gaps; *c*) advising on improvements to the ambient air quality monitoring network of the UAE; and *d*) conducting the first environmental epidemiologic study in the UAE.

The first three components of this work are described elsewhere (Davidson et al. 2011; Gibson 2011; Li et al. 2010; MacDonald Gibson and Farah 2012; Willis et al. 2010).

In this article we describe practical challenges of conducting the first environmental epidemiologic study in the UAE and how our research team overcame those challenges. Scientific details of the study are described elsewhere (Funk W, unpublished data; Ng et al. 2011; Yeatts et al. 2012). The epidemiologic study was designed to investigate exposures of the population to indoor air pollutants, resultant effects on health, and nutritional health status of residents.

We selected indoor air pollution as the focus of study, based on input from in-country representatives. During a fact--finding visit to the UAE, many people with whom we spoke expressed concern about dramatic changes in the built environment that have occurred in the UAE over the past genera-tion. Before the 1970s, most Emiratis were either nomadic or living in naturally ventilated reed huts on the coast. Air conditioning and tightly insulated buildings are new develop-ments, and adults who grew up in traditional structures asked us frequently whether they may be suffering ill health effects due to modern building practices. We focused our study on Emiratis. We did not include foreign workers, because of the transitory nature and large hetero-geneity of the foreign worker population, the lack of a reliable sample frame for foreign workers at the time, and the need for a tractable design for this initial epidemiologic pilot study. We acknowledge the limitations of an Emirati-only popula-tion as well as the need for additional future studies to examine the health of the expatriate work force in the UAE. Officials in UAE governmental agencies recognize the importance of including foreign workers in public health research and have taken action to address the issue. For example, government agencies in the UAE, such as the Ministry of Health, Health Authority–Abu Dhabi, Abu Dhabi Food Control Authority, and others, have initiated a study of environmental exposures in water and their potential effects on a study population in which foreign workers make up half of the targeted study sample. However, our study provided an opportunity to test methods and processes for conducting environmental epidemiologic research in the UAE, and it serves as a foundation on which future research can explore the impact of the environment on all who live and work in the Emirates.

Our study, completed in 2010, required the collective interdisciplinary efforts of 25 UNC and 50 United Arab Emirates University (UAEU) faculty and staff with assistance from EAD, the UAE National Bureau of Statistics, and the Health Authority–Abu Dhabi. We conducted a cross-sectional epidemiologic study in 628 Emirati households selected using a stratified random sample from national census data provided by the UAE National Bureau of Statistics (Yeatts et al. 2012). In each household, five gaseous pollutants (formaldehyde, hydrogen sulfide, sulfur dioxide, nitrogen dioxide, and carbon monoxide) and three particle-size fractions [particulate matter (PM) ≤ 2.5 µm (PM_2.5_), 2.5–10 µm (PM_2.5–10_), and ≤ 10 µm in aerodynamic diameter (PM_10_)] were measured over a 7-day period. We also collected health, dietary, stress, and physical activity data during home interviews with selected adults, adolescents, and children.

Practical lessons learned in this research can benefit others interested in conducting epidemiologic research in this understudied yet rapidly growing region. For successful research endeavors, cultural differences should be addressed and incorporated in all stages of the research process. We discuss our experiences with and recommendations related to the following issues: addressing stereotypes, data collection and study design, exposure measurements, schedules and time coordination, quality assurance and quality control, and institutional review board (IRB) protocol.

## Addressing Stereotypes

Many people have inaccurate, preconceived impressions of UAE culture and have questioned the ethicality of conducting research in the UAE. For example, some assume that Emiratis would discriminate based on sex, ethnicity, and religion and that Emirati nationals are disinterested in the large population of foreign workers. Before delving into the more detailed lessons learned, we believe it is important to address these inaccurate perceptions, which unfortunately are common.

Regarding issues of sex, female and male researchers were treated with equal respect. Many Emirati women hold advanced degrees; 51% of public university PhDs were awarded to women in 2010 (UAE National Bureau of Statistics 2010). Women, including the present Secretary General of the EAD, are leaders in many UAE government institutions. Our researchers included multiple members of the major religions, and all were treated respectfully. We did not observe or encounter discrimination based on skin color; we also noted that many Emirati citizens are of African descent. Finally, we found many Emirati citizens to be concerned about the welfare of the migrant workforce in the UAE, just as many U.S. citizens are troubled by mistreatment of migrant workers in the United States. In the focus-group sessions we held to gauge environ-mental health priorities of the local popu-la-tion, participants rated risks to workers in industry, agriculture, and construction as one of the three most important environmental health issues their nation is facing, along with outdoor and indoor air pollution (MacDonald Gibson and Farah 2012; Willis et al. 2010). One of the great benefits of this research was the opportunity to overcome our own pre-conceptions of UAE culture. We hope that many others will have similar experiences.

*Study and data collection design.* We encountered multiple cultural issues while designing the study and data collection protocols. Specific cultural knowledge was required to incorporate local customs in our household visit protocol, address appropriate inter-actions of men and women within our field staff teams, and modify our household-sampling procedures to include variations in family structure. For example, it was culturally inappropriate for male members of our field staff to enter the family common room or to interview women. More conservative female members of our teams of interviewers also considered it unacceptable to drive to a residential study site in the same vehicle with a male staff member. Study interviewers also encountered heads of households with more than one wife, each of whom lived in a separate household. In all cases, cultural awareness of societal norms was required.

Another concern in study design was seasonal migration of the UAE population. A substantial proportion of the Emirati popu-lation leaves the country during summer months to escape the intense heat. Such large-scale population migrations can compromise recruitment of households for study participation during the summer. Seasonality clearly impacts environmental studies that require year-round or summer assessment.

We also encountered rapid urban development and changes in national housing. Recent growth rates of major cities in the UAE are extraordinary; it is entirely feasible for neighborhoods to be constructed and populated in < 1 year. Equally feasible is the demolition of older neighborhoods, such as those with older governmental housing (shabias), and their complete reconstruction elsewhere in < 6 months. Thus, census data and population-sampling frames in the UAE quickly can become outdated.

Additionally, the study population’s overall unfamiliarity with research projects compared with many populations in the United States and Europe and the reluctance of families to be recruited via telephone presented challenges. Despite the endorsement of the government, publicity, and the distribution of letters in Arabic from the funding agency that described the study, some residents were suspicious of both the study interviewers and the air-monitoring equipment that would be placed in their household for a week.

Although the native language of our entire field staff was Arabic, language-related challenges also occurred. Some local dialects were difficult for field interviewers to understand; similar problems occurred with Arabic translation of data collection instruments. Appropriate formatting of the right-to-left orientation of Arabic script on the computer software used to conduct household surveys was also challenging.

**Recommendations.** We took several steps to address these cultural issues. First, all field staff teams included both men and women. For household visits, female interviewers were sex-matched with female family members and children, and male interviewers were matched with male family members. Male interviewers assembled in-home air monitoring equipment, whereas female inter-viewers placed equipment in the family common room. More conservative female interviewers drove separately to selected households and were reimbursed for their mileage. For heads of households with multiple wives, our local collaborators recommended that the most culturally sensitive solution was to invite each wife and her household to participate in the study and to adjust our household-sampling protocol accordingly. We also recruited additional local study staff to introduce research teams to participating households. We highly recommend this approach for similar future population-based studies.

To address seasonal migration of the popu-lation, we collected data throughout the fall, winter, and spring months, but not during the summer. We advise working with national and municipal agencies to obtain the most recent census information needed for population-based sampling. In our case, census data had been updated the previous year, which was sufficient for the majority of our selected population-sampling units. Upon finding neighborhoods that had been completely demolished to make way for new develop-ments, we worked with the national census agency to adjust our statistical sampling frame.

For household recruitment, our pilot study revealed that direct door-to-door contact was much more successful than telephone recruitment, because of tradition and the hospitable nature of the Emiratis. In accordance with the Bedouin culture of welcoming guests at the door, field teams often were greeted by residents with tea or coffee, snacks, or even a meal. In contrast, in the United States, it would be considered inappropriate in most cases to accept a meal. This culture of hospitality probably contributed to our higher-than-expected rates of participation, which at 75% is a notable strength of the study, especially given the demands on participants and multiple household visits.

To address Arabic language issues, Arabic versions of the study materials were reviewed and revised multiple times by UAEU colleagues familiar with local dialects. Training sessions were held with UAEU faculty and interviewer staff to review pronunciation of local Arabic words. To facilitate the production of Arabic language screens in the data management system, the entire computer screen was shown in a consistent Arabic format with English translations provided in optional pop-up screens. These measures greatly enhanced the ability of our field staff to gather the necessary data.

*Exposure assessment.* Assessing environmental exposures by passive monitoring of multiple household indoor air pollutants also was influenced by cultural factors. Our original sampling protocol required placement of monitoring equipment on a shelf or surface at breathing level (Funk W, unpublished data; Yeatts et al. 2012) in the room where the family spent the majority of their time. In accordance with the traditional nomadic lifestyle in the UAE, our pilot study revealed that some living rooms had cushions for sitting but little other furniture, which precluded sample deployment according to the protocol. In addition, we observed that households frequently had separate living rooms for men and women. These measurement protocol issues had to be addressed prior to the main study.

**Recommendations.** To standardize deployment of passive indoor air monitors in households, monitoring equipment was mounted on surveyor-grade tripods. We observed that the TV room was a common area shared by children and adults of both sexes. Thus, tripods were placed at a set height in the common living room. Sampling equipment was childproofed with screening material to prevent accidental tampering and was passive in design to minimize noise, disruption, and the need for power cords.

Outdoor air monitors were equipped with solar shields to protect equipment from intense sunlight and with manifolds to regulate air flow above the samplers. Outdoor tripods were stabilized with weighted buckets filled with sand or bricks to prevent toppling during dust storms.

*Schedules and time coordination.* We encountered several types of scheduling issues while conducting research in this region. The standard work week in the Middle East is Sunday through Thursday, compared with the Western tradition of Monday through Friday. On a routine basis, the work-week discordance compounds the 8- to 11-hr time zone difference between the Middle East and United States. These differences reduced the time our international team had together each week to collaborate.

On a yearly time scale, religious and govern-ment holidays are substantially different from the Western world. For religious holidays, Arab and other Muslim countries follow the Islamic lunar calendar in which each month begins with the sighting of the first crescent of a new moon. As a result, timing of holidays varies from year to year. During Ramadan, the holy month for Muslims, the population fasts during the day and eats and drinks only after sunset and before sunrise. In addition, a large proportion of the population in this region holds prayer five times a day, two of which occur during regular work hours. These prayer times can affect scheduled meetings and training sessions and require cultural awareness on the part of project staff.

In general, the concept of time in the Gulf region is more fluid than in the United States and Europe. This cultural difference should be carefully considered when conducting a project with a tight timeline and strict deadlines. Meetings and household recruitment visits frequently may be shifted or rescheduled on short notice. However, this perspective on time also allowed for greater flexibility in rescheduling meetings and household visits.

**Recommendations.** Differences in time zones, work week, religious observances, and practices required several adjustments in study protocol. Several U.S. project staff adopted the Middle Eastern work week during data collection. We recommend that all project leadership and staff receive basic education about religious norms and traditions of the study population as would be appropriate for any cross-cultural research project. Leadership and staff should consider the Middle Eastern work week, respect prayer times, and maintain a flexible approach to scheduling meetings and trainings. We also advise against conducting population-based research during the summer months or Ramadan in consideration of both the heat and the strict daily regime of religious observers, including study participants and in-country field staff.

## Quality Assurance and Quality Control

Because quality assurance and quality control (QA/QC) serve as a framework to assure the quality and confidence of the generated scien-tific data, it is essential that the entire study staff adhere to the protocols set forth in the QA/QC plan. Recruitment and hiring of field staff with scientific research experience and relevant expertise at all skill levels proved to be a major challenge. This issue was managed by in-depth staff training, as well as adherence and monitoring of the QA/QC protocol implementation.

**Recommendations.** Our QA/QC procedures were designed before the study and subsequently refined to maximize adherence to study protocols, data security, and confidentiality, and to address the lack of previous research experience by the field interviewers. Multiple feedback processes and checklists were designed and employed to catch errors made during data collection and correct them throughout the data collection phase.

For our data collection instrument, we used Data Management System (DMS), a computer-assisted personal interviewing instrument. This system was developed by the Collaborative Studies Coordinating Center (CSCC), UNC Department of Biostatistics (Chapel Hill, NC, USA), and has been used in > 30 multisite clinical trials and epidemio-logic studies. Interviewers used the DMS to collect an overall household census, individual participant data, measured air pollutant concentrations, and health questionnaire responses. Interviewers then uploaded data, securely transferred the data to the CSCC, and checked for data completeness and accuracy. Every 2 weeks, a series of data quality checks was conducted, and feedback was communicated to interviewers. We tracked household recruitment, interview completion status and the air monitoring data on a weekly basis. More details on the air pollutant QA/QC are provided elsewhere (Funk W, unpublished data; Yeatts et al. 2012).

Before data collection began, training and certification sessions for field interviewer staff were conducted in three major cities—Abu Dhabi, Dubai, and Al Ain. Training sessions were held in English, with Arabic translations offered to facilitate comprehension, because not all of the interviewers spoke fluent English. Whenever feasible, training materials included visual illustrations depicting study activities to reinforce training principles in a linguistically neutral fashion. All interviewers were trained in the use of the DMS, which included hands-on practice and a subsequent exam before they were certified and allowed to work on the study. On completion of training as well as data collection, certificates were distributed to the staff, who greatly appreciated acknowledgement of their efforts.

We also hired two UNC staff to reside in the UAE for the entire data collection period. One of their primary objectives was to help field staff consistently follow and implement data collection protocols on a daily basis. The staff also refined QA/QC protocols, which included systematic monitoring of all components of the study, including the indoor air, health, and nutrition sections.

## IRB Considerations

All research was conducted in accordance with UNC IRB and UAEU IRB approvals. The burden of combining multiple IRB protocol reviews has been noted by other researchers (Sly et al. 2009; Stenson et al. 2010). Although required by the IRB at our research university, minor protocol modifications, such as wording changes to the questionnaire for improved clarity or informed consent language simplification, were not standard practice in the UAE. Submission and review of these minor modifications added a significant load to IRB duties of our collaborators, and resultant delays greatly affected our in-country study timelines.

Cultural differences also influenced review of the informed consent agreement with household participants. Sly et al. (2009) and Stenson et al. (2010) discussed the difficulties of obtaining informed consent from study participants in developing countries where local ethical standards differ from global standards. The perception of the individual as autonomous is not necessarily valid in societies where a communal framework is the norm (Bhutta 2004; Stenson et al. 2010). These researchers cite an example in rural India where village elders made decisions for their entire community (Geller et al. 2006). We also encountered a few instances (< 1%) in the recruitment of our study families where the head of household refused study participation for female adults and children. We adhered to the basic principles of informed consent for family members. Family members were verbally informed of the study, and each family member had the option to decline participation, even if the head of household agreed to household participation.

**Recommendations.** Our UAEU colleagues contributed tremendously in facilitating in-country IRB reviews. These colleagues also advised us about the cultural importance of obtaining informed consent for all family members from heads of households. Our UNC IRB supported this culturally appropriate approach. Every field interviewer received training in human research ethics. It was critical to work collaboratively with our UAEU colleagues, receive guidance, and respond to feedback in designing our informed consent approach.

## Benefits and Lessons Learned

This multifaceted, international project benefited from the diverse professional and cultural backgrounds of its leadership and investigators. Our team included chemical engineers, statisticians, epidemiologists, econo-mists, industrial hygienists, environmental health scientists, computer programmers, dieticians, and nurses. Team members hailed from the UAE, United States, Sudan, Egypt, Jordan, Palestine, Kuwait, Lebanon, Somalia, Oman, and Tunisia. To work effectively, we built teams based on project-related tasks and used several linkage staff and faculty who facilitated communication and problem solving among both groups and disciplines. An overall project steering committee composed of project principal investigators and senior scientists provided leadership and a consensus vision to focus and integrate the three study components. Diverse thinking among the members of the many disciplines involved in the study sometimes posed challenges for communication but ultimately stimulated innovative thinking and novel solutions to field problems.

Projects of such interdisciplinary and national diversity require communication and compromise at all levels from senior leadership to field staff. For example, in study design, principal investigators must be willing to negotiate the level of effort and time devoted to their specific project relative to other complementary projects so the overall study objectives can be achieved. Such willingness to cooperate ensures not only the integrity of the study and its objectives but also the full commitment of all investigators. Net benefits of such multicultural and interdisciplinary approaches to complex environmental and social issues are holistic outcomes that are more readily interpreted and assimilated into problem resolution. In addition, net benefits of this cooperative teamwork included increased interdisciplinary and cross-cultural linkages among departments, institutions, faculty, and staff, establishing a basis for future research.

## Conclusions

Our experiences and lessons learned are germane to other areas of the world. Operationalizing cultural knowledge into study design and data collection protocols is critical when working with any population. Issues specifically relevant to this Arabian Gulf study include the need to incorporate aspects of regional culture into specific study logistics as well as the rapid economic transition of the study population that has led to enormous changes in the built environment and lifestyle. Research in this Gulf nation contributed to capacity building for the EAD staff and our entire project faculty and staff. We generated data and analyses that can be used for environmental and public health agency planning and policy decision making. As an interdisciplinary team, we benefited from team building within and among institutions. Our assessment of the environmental and health consequences that can occur in a country that has experienced rapid economic changes are scientifically applicable to other areas around the world. Additional benefits of conducting research in the UAE include the opportunity to develop good working relationships with government ministries and agencies and a renewed appreciation for the culture of hospitality that is widespread throughout the Middle East.

Cultural differences affect multiple aspects of an epidemiologic research study and should be addressed respectfully throughout the project. Challenges in the field can be met with careful planning, cultural sensitivity, and flexibility. Overall, the strong support for this research from EAD leadership, along with the culture of hospitality in the region and the dedication of the research staff from the involved institutions, led to the success of this research effort.

## References

[r1] Adams J, King C, Pendlebury D, Hook D, Wilsdon J (2011). Global Research Report Middle East: Exploring the Changing Landscape of Arabian, Persian, and Turkish Research.. http://researchanalytics.thomsonreuters.com/grr/.

[r2] Bhutta ZA (2004). Beyond informed consent.. Bull World Health Organ.

[r3] Davidson CA Krometis LA Al-Harthi SS Gibson JM 2011 Foodborne exposure to pesticides and methylmercury in the United Arab Emirates. Risk Anal; doi:10.1111/j.1539-6924.2011.01679.x [Online 6 October 2011] 21978365

[r4] Geller S, Naik V, Patel A, Goudar S, Edlavitch S, Kodkany B (2006). Ethical issues in conducting international research studies.. Int J Gynecol Obstet.

[r5] Gibson J. (2011). A burden of disease approach to prioritizing environmental policy initiatives: a case study in the Middle East.. N C Med J.

[r6] Li Y, Gibson JM, Jat P, Puggioni G, Hasan M, West JJ (2010). Burden of disease attributed to anthropogenic air pollution in the United Arab Emirates: estimates based on observed air quality data.. Sci Total Environ.

[r7] MacDonald Gibson J, Farah ZS (2012). Environmental risks to public health in the United Arab Emirates: a quantitative assessment and strategic plan.. Environ Health Perspect.

[r8] Ng SW, Zaghloul S, Ali H, Harrison G, Yeatts K, El Sadig M (2011). Nutrition transition in the United Arab Emirates.. Eur J Clin Nutr.

[r9] Sly PD, Eskenazi B, Pronczuk J, Sram R, Diaz-Barriga F, Machin DG (2009). Ethical issues in measuring biomarkers in children’s environmental health.. Environ Health Perspect.

[r10] Stenson AL, Kapungu CT, Geller SE, Miller S (2010). Navigating the challenges of global reproductive health research.. J Womens Health (Larchmt).

[r11] United Arab Emirates National Bureau of Statistics (2010). UAE in Figures 2010.. http://www.uaestatistics.gov.ae/ReportDetailsEnglish/tabid/121/Default.aspx?ItemId=1925&PTID=187&MenuId=2.

[r12] Willis HH, Gibson JM, Shih RA, Geschwind S, Olmstead S, Hu JH (2010). Prioritizing environmental health risks in the UAE.. Risk Anal.

[r13] Yeatts KB, El-Sadig M, Leith D, Kalsbeek W, Al-Maskari F, Couper D (2012). Indoor air pollutants and health in the United Arab Emirates (UAE).. Environ Health Perspect.

